# CARMA3: Scaffold Protein Involved in NF-κB Signaling

**DOI:** 10.3389/fimmu.2019.00176

**Published:** 2019-02-13

**Authors:** Shilei Zhang, Xin Lin

**Affiliations:** Department of Basic Medical Sciences, Tsinghua University School of Medicine, Beijing, China

**Keywords:** CARMA3, CARD10, NF-κB, GPCR, EGFR, DNA damage, virus infection

## Abstract

Scaffold proteins are defined as pivotal molecules that connect upstream receptors to specific effector molecules. Caspase recruitment domain protein 10 (CARD10) gene encodes a scaffold protein CARMA3, belongs to the family of CARD and membrane-associated guanylate kinase-like protein (CARMA). During the past decade, investigating the function of CARMA3 has revealed that it forms a complex with BCL10 and MALT1 to mediate different receptors-dependent signaling, including GPCR and EGFR, leading to activation of the transcription factor NF-κB. More recently, CARMA3 and its partners are also reported to be involved in antiviral innate immune response and DNA damage response. In this review, we summarize the biology of CARMA3 in multiple receptor-induced NF-κB signaling. Especially, we focus on discussing the function of CARMA3 in regulating NF-κB activation and antiviral IFN signaling in the context of recent progress in the field.

## Introduction

**Figure 1:**
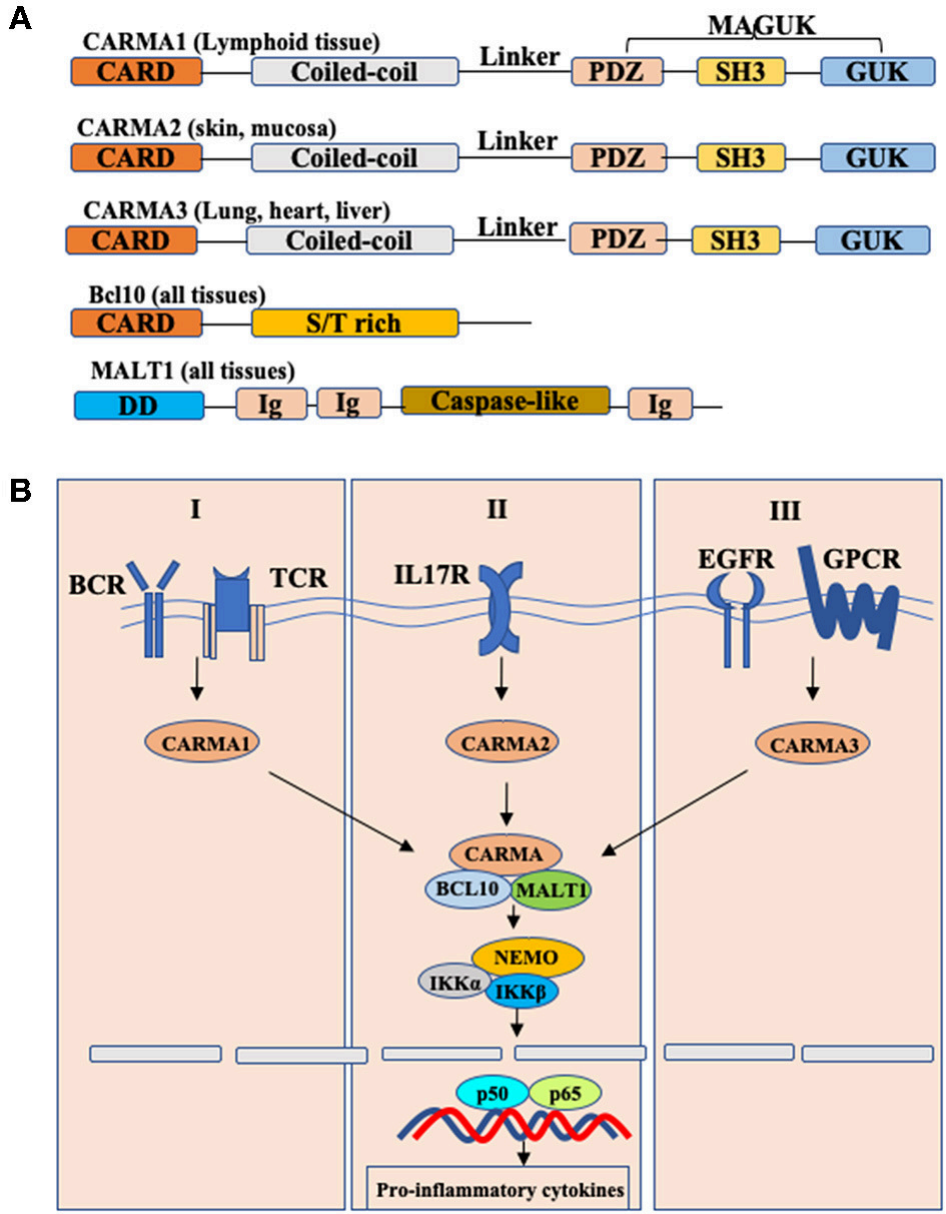
Schematic model of CBM complex-induced NF-κB activation. **(A)** Structures of CARMA1,2,3 and their interactors. Each CARMA member shares similar structures: caspase-recruitment domain (CARD), followed by a coiled-coil domain (CC), a linker region, a PDZ domain, an SH3 domain, and a GUK-like domain. BCL10, B-cell lymphoma/leukemia 10; S/T-rich, serine/threonine-rich region; MALT, mucosa-associated lymphoid tissue; DD, death domain; Ig, immunoglobulin-like domain; **(B)** Conserved Bcl10-Malt1 complexes interact with different CARMA proteins to link various receptors to NF-κB signaling.

Caspase recruitment domain and membrane-associated guanylate kinase-like protein 3 (CARMA3), also known as CARD10, is one of CARMA family members that include CARMA1, CARMA2, and CARMA3. CARMA proteins conserved across many species which are characterized by the different functional domains shared by all members of the family: the N-terminal CARD domain, following with a coiled-coil domain, a linker region, a PDZ domain, a SH3 domain, and the C-terminal guanylate kinase-like domain (GUK) (Figure 1) (1, 2). Although CARMA family proteins share a high degree of sequence and structural homology, they are transcribed by different genes and expressed in different tissues. Specifically, CARMA1 is primarily expressed in the hematopoietic system, including the spleen, thymus and peripheral blood leukocytes; CARMA2 is expressed in the mucosal tissues and skin; and CARMA3 is expressed in a broad range of tissues, particularly at high levels in lung, kidney, liver and heart, but not in hematopoietic cells (3).

CARMA proteins are also known as CARD11, CARD14, and CARD10, because they were originally identified as CARD domain-containing proteins by bioinformatics approaches. In spite of distinct tissue distribution, CARMA proteins mediate different signaling pathways but utilize a similar mechanism to activate downstream effector molecules (Figure 1) (4, 5). Upon signaling, CARMA proteins, through their CARD domain, recruit two signaling molecules: Bcl10 (B cell lymphoma protein 10), and MALT1 (mucosa-associated lymphoid tissue lymphoma translocation gene 1) to form the CARMA-Bcl10-MALT1 (also known as CBM complex), and then activate the downstream IKK complex, leading to the activation of NF-κB (6–8). Considering that Bcl10 and MALT1 are expressed in all tissues, the different tissue distribution indicates that CARMA proteins may function upstream of Bcl10 and MALT1 to mediate the certain receptor-induced NF-κB activation in different cells.

Early studies have showed that CARMA1 is required for antigen receptor-induced NF-κB activation in T cells and B cells, leading to lymphocyte activation and proliferation (9, 10). Recently, Wang et.al shows that CARMA2 plays a critical role mediating IL-17RA signaling in keratinocytes, and CARMA2 gain-of-function mutations result in constitutively activated IL-17RA signaling, leading to the development of skin inflammation and psoriasis (11). Previously, several studies indicate that CARMA3 functions as an indispensable adaptor protein in modulating NF-κB signaling downstream of some GPCRs (G protein-coupled receptors), including angiotensin II receptor and lysophosphatidic acid receptor, as well as receptor tyrosine kinases (RTKs), such as epidermal growth factor (EGF) receptor and insulin-like growth factor (IGF) receptor (12–14). Recent studies indicate that besides NF-κB signaling, CARMA3 also serves as a modulator in antiviral RLR signaling, providing a new understanding of CARMA3 (15). In this review, we summarize the biology of CARMA3 and discuss the roles of CARMA3 and its related proteins in different signaling pathways.

## Mechanism of CARMA3 Activation

Upon receptor activation, CARMA proteins may be recruited to the cytoplasmic membrane by adaptor proteins and then be further phosphorylated by upstream kinases, which results in the recruitment and activation of downstream effector proteins. In particular, in response to antigen receptors activation, PKCs are activated, which phosphorylate serine at S564/649/657 and S552 on the linker region of CARMA1, and triggers CBM complex oligomerization (16–19). Similar to CARMA1, phosphorylation of CARMA3 at Ser520, an analog to Ser552 of CARMA1, might be crucial for CARMA3 activation (16). Future studies are still needed to determine if other residues in CARMA3 can be phosphorylated by PKC, as well as other kinases may also contribute to CARMA3 activation. Upon phosphorylation, CARMA3 forms a CBM complex with Bcl10 and MALT1, contributing to downstream NF-κB activation by regulating the IKK complex activity through NEMO polyubiquitination (20).

Besides Bcl10 and MALT1, some other molecules are also reported to be involved in CARMA3 mediated NF-κB activation. D'Andrea et al. utilized a two-hybrid screening to identify a DEP domain-containing protein DEPDC7 as a cellular binding partner of CARMA2 and CARMA3 but not CARMA1, which serves as a specific mediator of GPCR-induced NF-κB activation (21). Upon GPCR activation, β-arrestin 2, but not the β-arrestin 1, associates with CARMA3 and most likely recruiting CARMA3 into the receptor complex to mediate LPA-induced NF-κB activation and subsequent IL-6 expression (22, 23). More recently, Jiang et al. identified a transmembrane protein, TMEM43 (also known as LUMA) as a new CARMA3-associating protein that contributes to EGFR-induced NF-κB activation in cancer cells (24).

## CARMA3 in EGFR Signaling and GPCR Signaling

**Figure 2:**
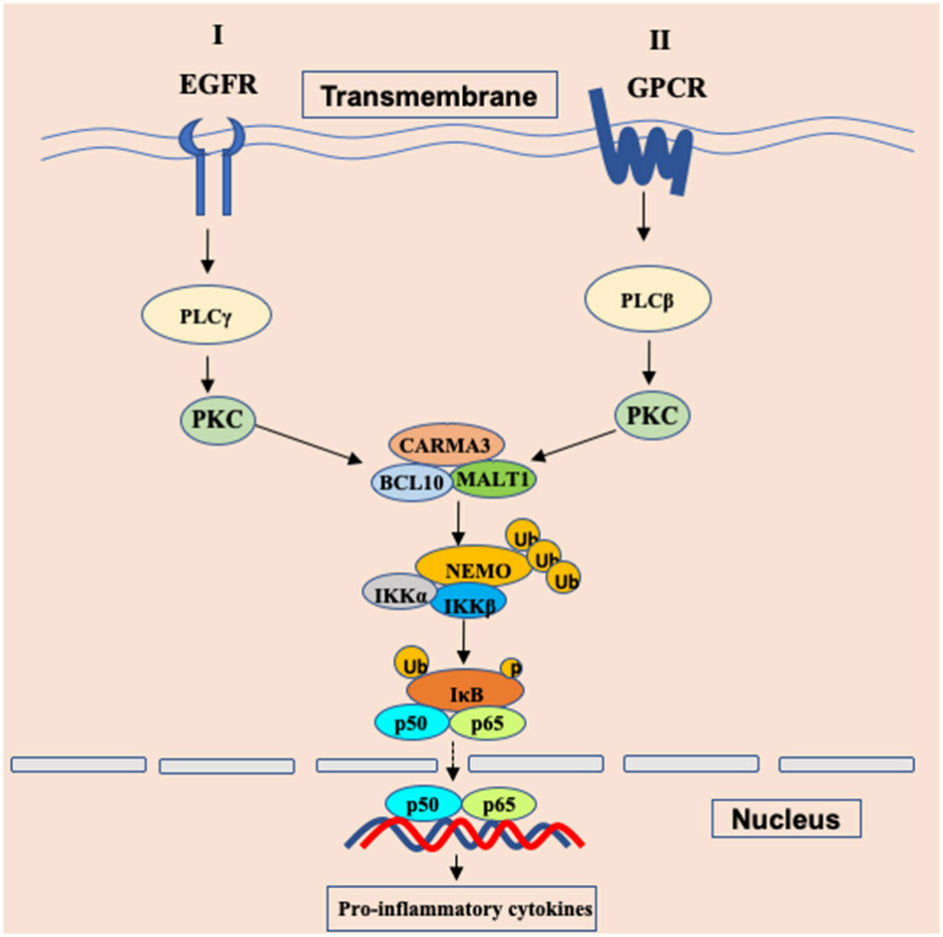
Schematic of GPCR/EGFR-CARMA3 signaling pathway leading to NF-κB activation. In cancer cells and endothelial cells, stimulations of the surface receptor EGFR and GPCR trigger the proximal signaling, leading to activation of phospholipase C (PLC) and protein kinase C (PKC). The activated PKC further phosphorylates CARMA3 and enables CARMA3 to form the CBM complex through interaction with the downstream Bcl10 and MALT1. Formation of the CBM complex leads to activation of the IKK complex through NEMO polyubiquitination. Ub, ubiquitination; p, phosphorylation.

Since receptor tyrosine kinases (RTKs), integrins, and G protein-coupled receptors (GPCRs) have been reported to activate NF-κB signaling through PKCs (14), Grabiner BC et al. found that CARMA3-deficient mouse embryonic fibroblasts (MEFs) failed to trigger NF-κB activation upon stimulation with endothelin (ET-1) (20) and lysophosphatidic acid (LPA) (20), which are ligands for two different GPCRs. Specifically, CARMA3 is mainly required for GPCR-induced NF-κB activation (Figure 1), because this activation induced by other cell surface receptors such as TNFR or TLR4 do not require CARMA3 (20). Similar to CARMA1 in antigen receptor-induced NF-κB activation, CARMA3 also controls NF-κB activation by forming the CBM complex. Several laboratories showed that GPCR-induced NF-κB activation is also defective in Bcl10-deficient cells (25, 26). In addition, Malt1 is also critically required for the degradation of IκBα and the subsequent NF-κB induction in response to LPA stimulation (27, 28). Moreover, Bcl10 and Malt1 are selectively for LPA-induced NF-κB activation but are dispensable for the activation of the JNK, p38, ERK MAP kinase, and Akt signaling pathways (25). By analyzing angiotensin II (Ang II)-induced NF-κB activation, blocking the function of any components in the CARMA3-Bcl10-Malt1 signalosome, through the use of either RNAi, dominant-negative mutants, effectively impairs Ang II-induced NF-κB activation (27). In endothelial cells, the angiotensin II receptor AGTR1 induces NF-κB-dependent pro-inflammatory responses, which is relied on PKC-dependent assembly of a signaling complex comprised of CARMA3, Bcl10, and MALT1, contributing to endothelial dysfunction and vascular disease (29–32). In some breast cancers, aberrantly overexpressed AGTR1 induces both ligand-dependent and ligand-independent NF-κB activation, mediated by CARMA3, Bcl10, and MALT1, driving cancer cell-intrinsic responses that include proliferation, migration and invasion, as well as cancer cell-extrinsic effects to promote tumor angiogenesis through impacting endothelial cells of the tumor microenvironment (33). In airway epithelial cells (AECs), Causton et al. showed that CARMA3 contributed to NF-κB activation and the production of proasthmatic mediators in response to a panel of asthma-relevant GPCR ligands. Through genetically modified mice with CARMA3-deficient AECs, they demonstrated that CARMA3 in AEC is involved in allergic airway inflammation and bridges the innate and adaptive immune responses in the lung (34, 35). More studies have further indicated that the CARMA3-Bcl10-Malt1 signalosome plays a critical role in other GPCRs-induced NF-κB activation in different cellular context (27, 33, 36, 37). Together, these studies suggest that CARMA3-Bcl10-Malt1 signalosome is an essential signaling complex linking GPCRs to NF-κB activation (Figure 2).

Receptor tyrosine kinases (RTKs), a family of cell surface receptors, are key players in mediating multiple cellular responses upon the stimulation of growth factors, cytokines, and polypeptide hormones. Multiple growth factors, including insulin-like growth factor (38), epidermal growth factor (39), and fibroblast growth factor (40), can induce notable NF-κB signaling through their receptors that belong to the RTKs family. Since PKC is required for EGFR-induced NF-κB activation and given the importance of CBM complex in IKK activation (14), Jiang et al. explored a possibility whether EGFR-induced NF-κB activation involves the CBM complex. Consistent with this hypothesis, their data indicate that CARMA3 and Bcl10 are required for EGFR-induced NF-κB activation in both EGFR-expressing human cancer cell lines and mouse embryonic fibroblasts (41). Using biochemical and genetic approaches, Pan et al. found that CBM complex is required for HER2-induced NF-κB activation and functionally contributes to multiple properties of malignancy, including proliferation, migration and invasion, both *in vitro* and *in vivo* (42). Upon stimulation, the CBM complex recruits E3 ligase TRAF6 to provide a positive signal to activate IKK complex via Lys63-linked polyubiquitination (43). Therefore, these studies indicate that stimulation of RTKs, including EGFR and HER2, induces NF-κB activation through CARMA3-associated complex (Figure 2).

However, it remains to be determined the mechanism by which the CBM complex is linked to EGFR signaling pathway. To address this question, Jiang et al. performed a high-throughput and functional genomic screen (also named Bi-molecule fluorescence complementation assay, BiFC) to identify potential CARMA3-binding proteins, and found that TMEM43 (also known as ARVD5 or LUMA) might be a critical component in EGFR signaling network through interaction with CARMA3 and its associating complex to induce downstream NF-κB activation following EGF stimulation, but not on TNF-α stimulation (24). Mechanically, they revealed that TMEM43 inducibly interacted with EGFR and mediated the formation of CARMA3 and Bcl10 complex (24), which may bridge EGFR and CBM complex, leading to the activation of IKK. Regarding to the intracellular protein tyrosine kinase activity of EGFR, it still need to be investigated whether the inducible association between EGFR and TMEM43 may lead to TMEM43 or its binding proteins being phosphorylated by EGFR, which may enable TMEM43 to interact with CARMA3/Bcl10 complex.

## Contribution of CARMA3 to DNA Damage Response

**Figure 3:**
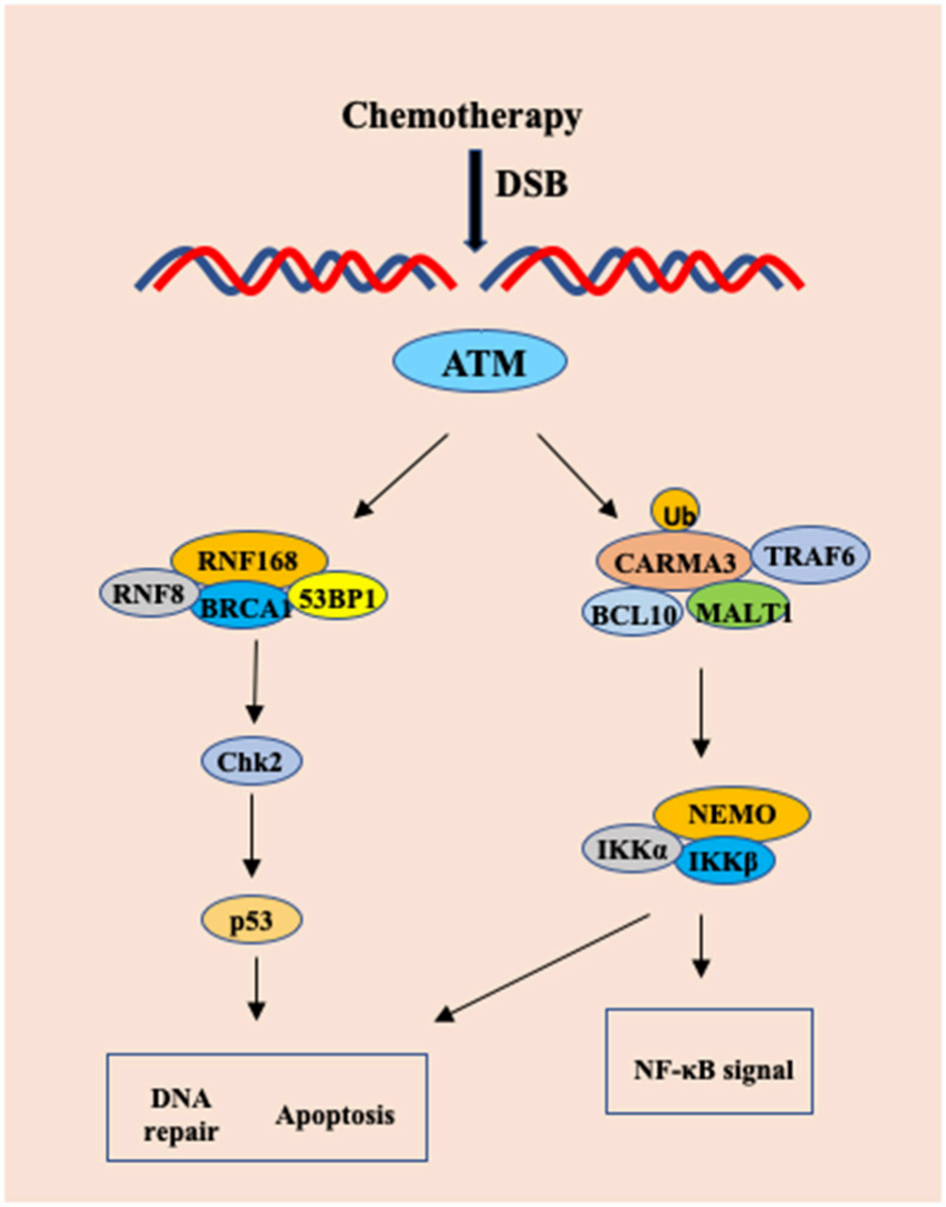
The CARMA3-BCL1-MALT1 (CBM) complex contributes to DNA damage-induced NF-kB activation. Upon chemotherapy drug in HeLa cells and MEF cells, DNA damage-induced NF-kB activation involves the recruitment of TRAF6 to CARMA3, which leads to formation of the CARMA3/Bcl10/MALT1/TRAF6 complex, which finally results in a CARMA3-dependent, but PKC-independent polyubiquitination of IKK complex.

NF-κB is a family of transcription factors that induce the expression of multiple anti-apoptotic genes (44, 45). Although NF-κB is activated as part of DNA damage responses, which protects cells from DNA damage-induced program cell death (46), the molecular mechanism by which DNA damage activates NF-κB remains to be determined. Zhang et al. showed that the CBM complex not only responds to extracellular stimuli by various receptors, but also mediates intracellular signal elicited from ATM-mediated DNA damage response (47). PKC is generally considered regulating CARMA1 or CARMA3 activation through phosphorylation of the linker region of CARMA proteins (17, 48). However, Zhang et al. found that PKC activity is dispensable for Doxorubicin-induced NF-κB activation (47), indicating that other kinases may phosphorylate CARMA proteins, or alternatively, CAMRA proteins may be activated through different mechanisms, such as ubiquitination upon DNA damage. Interestingly, this study revealed that CARMA3 could be modified by K63-linked ubiquitination upon Doxorubicin stimulation (47). In addition, CBM complex is linking TRAF6 to IKK complex in response to EGF stimulation in A431 cells (49). Consistent with the role of TRAF6 in activation of IKK, Zhang et al found that DNA damage triggered the association between CARMA3 and TRAF6, suggesting that TRAF6 may function to activate CBM complex by K63-linked polyubiquitination of CARMA3 (Figure 3) (47).

The anti-tumor effect of many chemotherapy drugs relies on their ability to induce cell apoptosis due to the DNA damage response (50, 51). However, cancer cells can induce NF-κB activation as a mechanism to avoid DNA damage-induced apoptosis and develop drug resistance for chemotherapy (52). Since Doxorubicin induces more cell apoptosis in CARMA3- and Bcl10-deficient cells than WT cells, this result suggests that the CBM complex might contribute to the resistance of chemotherapy-induced cell death (47). Although irradiation-induced damage can be repaired, damage to normal tissue is one of main side effects for cancer radiotherapy. In their study, Zhang et al. found that the tissue repair and cell proliferation were impaired in CARMA3-deficient mice exposed to irradiation, indicating the protective role of CARMA3 in cell survival (47). Together, this study revealed the molecular mechanism by which DNA damage activates NF-κB is mediated by CBM complex, therefore, providing a molecular basis for targeting the CBM complex to block DNA damage-induced NF-κB pathway (47).

## Role of CARMA3 in Antiviral RIG-I-MAVS Signaling

**Figure 4:**
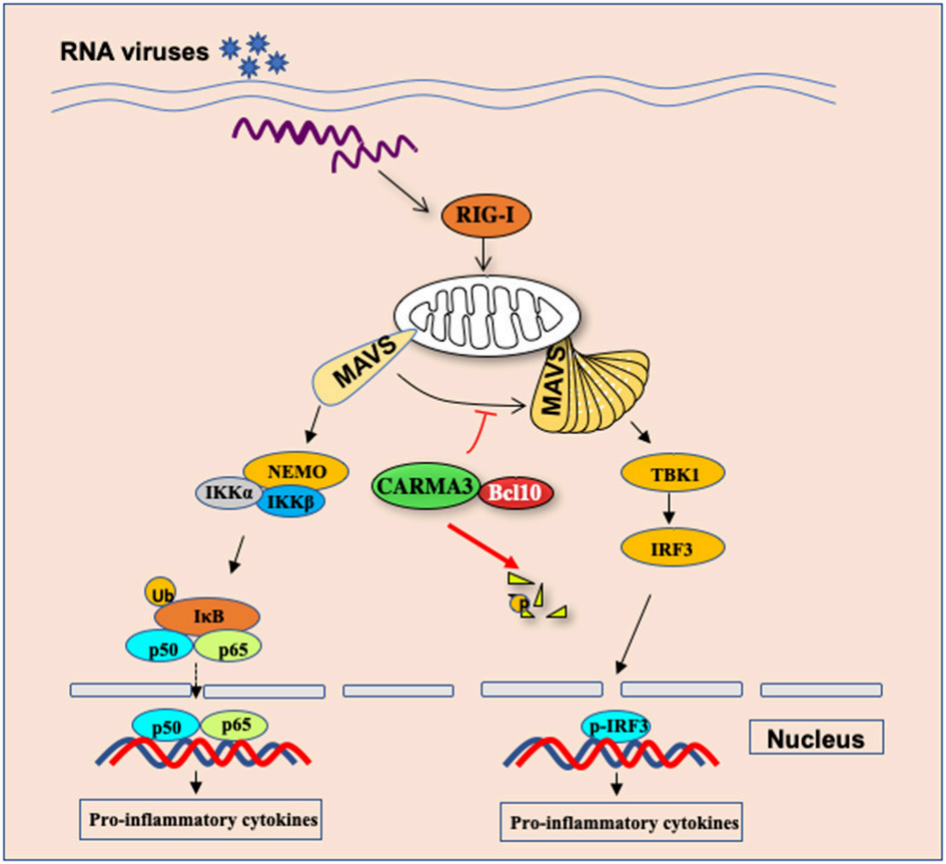
CARMA3 Is a host factor regulating the balance of inflammatory and antiviral responses against viral infection. Upon RNA virus infection in airway epithelial cells, CARMA3 sequesters MAVS from forming high-molecular-weight aggregates, thereby suppressing TBK1/IRF3 activation. Following NF-kB activation upon virus infection, CARMA3 undergoes K48-linked ubiquitination and is targeted for proteasome-dependent degradation, which releases MAVS to activate IRF3.

It has been an important question why different individual displays highly variable responses and infectious outcomes to influenza virus infection. To explore the host genetic polymorphisms contributed to this variation, Ferris et al. identified quantitative trait loci (QTL), Hrl4, contains 13 genes (53). Among of these genes, Card10 gene, which encodes CARMA3, may have a potential link to the antiviral innate response. To determine the functional role of CARMA3 in antiviral innate immune response, Jiang et al. challenged CARMA3-deficient mice with influenza virus or vesicular stomatitis virus and found that CARMA3-deficient mice showed more resistance to virus infection, which was characterized by less weight loss, lower viral yield, and greatly attenuated lung injury, suggesting that CARMA3 plays a negative role in antiviral response against virus infection (15). Transcriptional factors NF-κB and IRFs induce the expression of multiple antiviral pro-inflammatory cytokines and type I interferon expression. Interestingly, following influenza virus or vesicular stomatitis virus infection, the production of pro-inflammatory cytokines, such as IL-6, IL-1β, and TNF-α in the lung or sera of CARMA3-deficient mice was significantly reduced compared to wild-type mice, while more type I IFN was induced in CARMA3 KO mice, indicating that CARMA3 plays a negative role in regulating antiviral responses in the host, but it plays a positive role in regulating the expression of pro-inflammatory cytokines in response to viral infection (Figure 4) (15). Mechanically, they found that CARMA3 is a regulator of RIG-I-MAVS signaling pathway, which modulates MAVS-mediated NF-κB and TBK1-IRF3 activation in a two-phase mechanism (15). Upon RIG-I activation at the early time of viral infection, MAVS is firstly activated on mitochondrial, activating downstream IKKα/IKKβ/NEMO signaling in a CARMA3-dependent manner (Figure 4). However, in the early phase of post-infection, the CARMA3-BCL10 complex interacts with MAVS, therefore, preventing the formation of high-molecular weight MAVS aggregates that is required for downstream TBK1-IRF3 activation (Figure 4). Unlike other reported molecules including A20, CYLD, and NEMO, which are either positive or negative mediators for both TBK1-IRF3 signaling and NF-κB activation (54–56), CARMA3 contributes to RNA virus infection-induced NF-κB signal but prevented TBK1/IRF3 activation through disruption of MAVS oligomerization. However, RNA virus infection also activates an unknown E3 ubiquitin ligase that induces CARMA3 polyubiquitination and degradation (Figure 4), leading to releasing the CARMA3-dependent inhibition on the MAVS-TBK1-IRF3 signaling in the late phase of RNA virus infection (15).

Since CARMA1, instead of CARMA3, is expressed in hematopoietic cells, it will be interesting to determine whether CARMA1 functions in mediating RIG-I/MAVS signaling in myeloid cells. However, our unpublished data revealed that CARMA1 was not involved in the regulation of neither RIG-I/MAVS in anti-RNA virus nor cGAS/STING in anti-DNA virus signaling (data was not shown here). In addition to CARMA1, CARD9, a protein structurally related to CARMA family but only expressed in myeloid cells, resulted in defects in NF-κB activation and production of pro-inflammatory cytokines, including IL-6 and IL-1β, in response to 5′ppp dsRNA treatment and DNA virus infection (57, 58). However, it did not alter the production of type I IFN. In contrast to the CARMA family that are differentially expressed in different tissues and cells, BCL10 is ubiquitously expressed. Similar to the role of CARD9 in dendritic cells, BCL10 mediated NF-κB activation and production of pro-inflammatory cytokines in response to 5′ppp dsRNA treatment or DNA virus infection (57, 58), whereas, in primary MEF cells, BCL10 functions similarly to CARMA3 upon VSV infection or poly(I:C) treatment (15), suggesting that BCL10 may regulate RIG-I/MAVS signaling in a cell-type-specific manner.

During the past decade, it has been shown that posttranslational modifications including phosphorylation, ubiquitination, and SUMOylation, play important roles in fine-tuning innate immunity by either modulating the stability of key proteins in the immune system (59). Recent studies indicate that ubiquitination may also regulate the function of CBM complex. Several laboratories showed that in lymphocytes, TCR activation induces the ubiquitination of lysine residues in the SH3 and GUK domains of CARMA1, leading to a proteasome-dependent degradation of CARMA1; while in natural killer T cells, the E3 ligase Cbl-b mediated the agonistic ligand α-galactosylceramide-induced ubiquitination and degradation of CARMA1 (60, 61). Similar processes also regulate BCL10 as shown that PKC or TCR/CD28 co-stimulation signaling in primary T cells results in ubiquitination of Bcl10 and degradation by the autophagy-dependent proteolysis machinery but not by the proteasome complex (61). Thus, these studies indicate a feedback mechanism in which E3 ubiquitin ligases mediate ubiquitination and degradation of CARMA1-BCL10 complex following the stimulation of antigen receptors. However, whether the CARMA3-BCL10 complex also undergoes ubiquitination and proteasomal degradation is still unclear. Jiang et al. revealed that the stability of BCL10 was not significantly altered following VSV infection, indicating that posttranslational modification of BCL10 is a signaling-specific manner. In contrast to BCL10, CARMA3 is gradually targeted for K48-linked ubiquitination and degradation following VSV infection (15), which may serve as a new mechanism to attenuate NF-κB signaling, and meanwhile, it releases MAVS from CARMA3-BCL10-MAVS complex to form functional aggregates to trigger TBK1-IRF3 activation (Figure 4) (15). Altogether, it suggests that CARMA3 is a key factor that regulates the balance of inflammatory and antiviral responses against viral infection. However, it remains to be determined how CARMA3 is targeted for K48-linked ubiquitination and degradation, which may help to design therapeutic agents for reducing inflammation but enhancing the antiviral response.

## Conclusions and Outstanding Questions

During the past decade, many progress has been made in the understanding of CARMA3 functions in the NF-κB signaling pathways. These studies demonstrate that CARMA3-dependent IKK activation is involved in GPCR-, RTK-, ATM-, and RLR-induced NF-κB activation. However, further investigation is still needed to reveal the mechanism by which CARMA3 are linked to the different receptors. Therefore, it is important to determine whether CARMA3 is associated with other proteins following different stimuli. Identifications of such proteins will provide the molecular basis of how CARMA3-containing complexes are involved in mediating NF-κB activation induced by different stimuli.

Several groups have shown that CARMA3 was overexpressed in several tumor cells and correlated with tumor progression (42, 62, 63), indicating that CARMA3 may serve as a potential drug target for cancer treatment. Moreover, Zhang et al. highlights the role of CARMA3 in DNA damage-induced NF-κB activation, which may explain the resistance of some tumor cells to chemotherapy or radiation-therapy (47). Thus, more studies are required to define the regulation of CARMA3-mediated signaling transduction and the role of CARMA3 or its interacting proteins in cancers.

Given that *Card10*, CARMA3 encoding gene, might contribute to the host susceptibility to influenza virus (53), and since CARMA3 plays a positive role in RIG-I-induced NF-κB activation, leading to the induction of pro-inflammatory cytokines, but negatively regulates MAVS-induced TBK1/IRF3 signaling and production antiviral Type-I IFN (15), it will be interesting to investigate the role of CARMA3 in influenza virus pathogenesis. Considering the ubiquitination and degradation of CARMA3 upon VSV infection, it will be interesting to determine whether some virus, such as influenza virus, Ebola, SARS, and MERS, may modulate the mechanism of proteasomal degradation of CARMA3, which may regulate the expression of pro-inflammatory cytokines and type I IFN expression, and result in virus infection-associated cytokine storm, thereby contributing to the pathogenesis of virus infection.

Until now, no missense mutations of CARMA3 have been reported to contribute to the pathogenesis in human disease, besides some studies suggest that CARMA3 is overexpressed in several tumors (63), which may affect the onset and progression of tumorigenesis. Therefore, it will be interesting to investigate whether there are CARMA3 polymorphisms that affects the ubiquitination and degradation of CARMA3, resulting in variations of individuals to tumor development, chemotherapy resistance, and susceptibility to virus infection. This kind of study will provide the new molecular insight for designing the therapeutic agents for cancer and infectious diseases.

## Author Contributions

All authors listed have made a substantial, direct and intellectual contribution to the work, and approved it for publication.

### Conflict of Interest Statement

The authors declare that the research was conducted in the absence of any commercial or financial relationships that could be construed as a potential conflict of interest.
